# Influence of icariin on inflammation, apoptosis, invasion, and tumor immunity in cervical cancer by reducing the TLR4/MyD88/NF-κB and Wnt/β-catenin pathways

**DOI:** 10.1186/s12935-021-01910-2

**Published:** 2021-04-13

**Authors:** Chunyang Li, Shuangqing Yang, Huaqing Ma, Mengjia Ruan, Luyan Fang, Jing Cheng

**Affiliations:** 1 Department of Gynecology and Obstetrics, Reproductive Center, the Second Affiliated Hospital and Yuying Children’s Hospital of Wenzhou Medical University, Wenzhou, Zhejiang China; 2 Department of Biochemistry, School of Basic Sciences, Wenzhou Medical University, Wenzhou, Zhejiang China

**Keywords:** Icariin, Cervical cancer, Inflammation, Tumor immunity, Microbiota

## Abstract

**Background:**

Cervical cancer is a type of the most common gynecology tumor in women of the whole world. Accumulating data have shown that icariin (ICA), a natural compound, has anti-cancer activity in different cancers, including cervical cancer. The study aimed to reveal the antitumor effects and the possible underlying mechanism of ICA in U14 tumor-bearing mice and SiHa cells.

**Methods:**

The antitumor effects of ICA were investigated in vivo and in vitro. The expression of TLR4/MyD88/NF-κB and Wnt/β-catenin signaling pathways were evaluated.

**Results:**

We found that ICA significantly suppressed tumor tissue growth and SiHa cells viability in a dose-dependent manner. Also, ICA enhanced the anti-tumor humoral immunity in vivo. Moreover, ICA significantly improved the composition of the microbiota in mice models. Additionally, the results clarified that ICA significantly inhibited the migration, invasion capacity, and expression levels of TGF-β1, TNF-α, IL-6, IL-17A, IL-10 in SiHa cells. Meanwhile, ICA was revealed to promote the apoptosis of cervical cancer cells by down-regulating Ki67, survivin, Bcl-2, c-Myc, and up-regulating P16, P53, Bax levels in vivo and in vitro. For the part of mechanism exploration, we showed that ICA inhibits the inflammation, proliferation, migration, and invasion, as well as promotes apoptosis and immunity in cervical cancer through impairment of TLR4/MyD88/NF-κB and Wnt/β-catenin pathways.

**Conclusions:**

Taken together, ICA could be a potential supplementary agent for cervical cancer treatment.

## Background

Cervical cancer, one of the gynecological cancers, is a malignancy with a frequency increasing year by year, and it is widely recognized to be the outstanding cause of cancer death in females [1]. This cancer is caused by infection with high-risk human papillomavirus (HPV) at the junction of cervix vaginal or transitional band scale-columnar epithelial cells [2]. Most of the patients were diagnosed with advanced or recurrent cancer since the tumor has characteristics of metastatic and poor prognosis [2]. Chemotherapy based on cyclophosphamide and cisplatin has been considered to be the first-line treatment for cervical cancer, which is due to the high survival rate after treatment [3]. However, the clinical application of chemotherapy drugs was limited due to subsequent risks of induced drug resistance, bone marrow suppression, nerve damage, and other severe adverse reactions [4]. Due to the advantages of traditional Chinese medicine (TCM), such as precise curative effect, low toxicity side effect, and multi-target, the clinical application of TCM has become popular worldwide. According to previous studies, TCM has demonstrated significant inhibition on the tumor, thus, TCM was of considerable interest in cervical cancer treatment [5].

*Epimedium* (Berberidaceae), a genus of herbaceous plants with 52 species, is used in TCM for thousands of years. This genus was documented to be remarkable therapies for multiple diseases, especially in sexual dysfunction, osteoporosis, and immune disorders. The major components of *epimedium* extracts are flavonoids, and among them, icariin (ICA, C_33_H_40_O_15_, molecular weight: 676.67) is detected to be an active chemical component. The function of ICA has been shown to regulating endocrine systems, improving cardiovascular function, strengthening immune systems and neuroprotective activities. Besides, it was investigated to possess an anti-tumor effect, which mainly focuses on inhibiting the proliferation of tumor cells and the formation of tumor blood vessels, inducing tumor cell differentiation and apoptosis, changing cell cycle distribution, blocking inflammatory signaling pathways, and inhibiting telomerase activity [6]. According to oncology and immunology studies, the occurrence and development of malignant tumors were significantly considered with tumor-cell immunity. ICA has been thought to improve the immune organs and cells, enhance the activity of immune cytokines, to facilitate the regulation of tumor immunity. Meanwhile, increasing researcher awareness of TCM and reports demonstrated that treatment with TCM might cause changes in gut microbiota [7].

In the gut of all mammals, there is a micro-community consist of bacteria, fungi, viruses, and protozoa, known as gut microbiota. This colony plays an important role in the immunity system, physiological states, and many metabolic processes, which include digestion, absorption, and nutrition. The development of a tumor is regulated by multiple cellular and non-cellular components in the tumor microenvironment (TME), such as immune cells, cytokines, chemokines, and related signaling pathways. Whereas, the imbalance of gut microbiota will inactivate the inflammatory pathway, and lead to changes in TME, which may promote the development of a tumor and inhibit the monitoring of the immune system [8]. In the innate immune system, toll-like receptors (TLRs) acts on the initiation of inflammatory defense mechanisms, but it can also cause chronic inflammation in unhealthy tissues, providing a favorable environment for tumor growth [9]. Recent studies suggested that the overexpression of the TLR4 was correlated with immune escape and apoptosis resistance in cervical cancer. Besides, TLR4 can bind with MyD88 adapters, leading to activation of nuclear factor-kappa B (NF-κB), which results in the pro-inflammatory response [10, 11]. Accumulating evidence indicated that the Wnt/β-catenin pathway possessed a significant effect on cell growth, proliferation, invasion, and apoptosis. Signals were transmitted by β-catenin to trigger the downstream target gene expression, and β-catenin regulates the development of tumor cells after combining with the E-cadherin on cell membranes. Therefore, the aberration of Wnt /β-catenin has been considered as a factor of tumorigenesis [12]. However, the effect of ICA on the deregulated signaling pathway remains unclear.

The current study aimed to explore a medical basis for cervical cancer by performing U14 tumor models in nude mice and treating them with different concentrations of ICA, analyzing the proliferation, migration, invasion, and apoptosis of tumor cells. The correlations between ICA and expression levels of TLR4/MyD88/NF-κB and Wnt /β-catenin signaling pathway were observed, for purpose of providing novel insight into cervical cancer.

## Materials and methods

### Cell lines and cell culture

The cervical cancer cell lines U14 and SiHa were obtained from American Type Culture Collection (ATCC, Rockville, MD, USA) and cultured respectively in RPMI 1640 (Gibco, Carlsbad, CA) and Dulbecco’s modified Eagle’s medium (DMEM, Sigma-Aldrich, St. Louis, MO, USA) supplemented with 10% fetal bovine serum (FBS) with antibiotics in a humidified incubator with 5% CO_2_ atmosphere at 37 °C.

### Experimental animals and treatment

A total of 75 SPF female Kunming mice (4 weeks old, 20–22 g) were purchased from the Shanghai SLAC Laboratory Animal Co., Ltd. (Certificate No. SCXK (Hu) 2013–0018; Shanghai, China) and were housed in Hangzhou Eyong Biotechnological Co., Ltd. Animal Experiment Center (Certificate No. SYXK (Zhe) 2020–0024) with a 12 h light: 12 h dark cycle, temperature 20–25 °C, humidity 50–65%, and had free access to standard chow and tap water. The 75 mice were divided into 3 batches, with 25 mice in each batch. Single-tumor U14 cell suspension (1 × 10^6^ cells in 0.1 mL PBS) was injected subcutaneously into the right flank of each mice to obtain xenograft tumors. The body weight and tumor growth of the mice were recorded weekly. Also, the volume of the tumor reached 150 mm^3^ after cell implantation, the mice were randomly divided into five groups (5 mice per group): Model group, cyclophosphamide (CTX) group, low-dose ICA group (ICAL), medium-dose ICA group (ICAM), and high-dose ICA group (ICAH). Mice in the ICAL, ICAM, ICAH, and CTX groups were intragastrically via oral gavage administered the ICA (CAS NO. 489–32-7, medical-grade, ≥ 96.0%, Aladdin, China) at the dose of 10, 20, 40 mg/kg body weight per day, and CTX (25 mg/kg body weight per day, Millipore Sigma, St. Louis, MO), respectively. Mice in the model group received an equal volume of normal saline. The tumor dimensions were measured using digital calipers daily, and the tumor volume(V) was calculated by tumor length (L) and width (W) as V = L × W^2^/2. The mice were anesthetized with 5% ether after 28 days, and the body weight of the third batch of mice was recorded, as well as the tumor samples were removed and weighed and then fixed in 10% formalin for an investigation on the pathological changes. Subsequently, the spleen and thymus of the mice were isolated, and the excess tissues and fascia were stripped and then weighed. Besides, the spleen and thymus indexes were calculated according to the following equation: organ index = organ mass/body weight. Additionally, the anti-tumor rate was calculated as follows: (1-average weight of tumor in the treatment group/average weight of tumor in model group) × 100%. Animal experiments were approved by the ethics committee of Hangzhou Eyong Biotechnological Co., Ltd. Animal Experiment Center. All the mice were treated based on internationally accepted ethical procedures.

### Carbon clearance test

The first batch of mice was employed in the carbon clearance test, which was used only in this batch as it would interfere with the results of other tests in the current study. After the final administration for 30 min, the phagocytosis was evaluated in vivo by the carbon clearance trial. Briefly, carbon ink diluted with saline injections (dilution 1:10) was injected into mice (0.1 ml per 10 g body weight) by a single tail vein injection. 20 μLblood samples were collected from the canthus venous plexus at the 1 min and 7 min after ink injection and were subsequently added to 2 ml of 0.1% Na_2_CO_3_. After that, the absorbance (OD) of the solutions was measured at 600 nm by using MD SpectraMax M5 microplate reader (Molecular Devices, USA). Later on, all the mice were sacrificed, then the spleen and liver were rapidly removed and weighed. Finally, the phagocytic index (K) and phagocytic activity (a) were calculated respectively using the following formula:

$$\eqalign{ & K = \left( {lo{g_{10}}O{D_1} - lo{g_{10}}O{D_2}} \right)/\left( {{T_{7min}} - {T_{1min}}} \right);{\text{ }} \cr & a = {\text{ }}\left[ {\left( {body{\text{ }}weight} \right)/\left( {{\text{ }}spleen{\text{ }}weight + liver{\text{ }}weight} \right)} \right] \times \sqrt[3]{K} \cr}$$

### Measurement of serum hemolysin

The second batch of mice was used to perform the serum hemolysin test. Briefly speaking, the mice were intraperitoneally injected with 0.2 ml 5% SRBC for immunization. After 5 days, the mice were anesthetized with 5% ether and the blood samples were collected by removing their eyeballs, and then the serum was obtained by using the centrifugation method at 3000 rpm/min for 15 min. After that, the serum was diluted 100 times with normal saline, and then the diluted serum was mixed with 0.5 ml 10% complement solution (Nanjing SenBeiJia Biological Technology Co., Ltd., Nanjing, China), 0.5 ml 5% SRBC suspension, and 0.5 ml saline. The control was set as 50% hemolysis (0.5 ml 5% SRBC suspension with 1.5 ml normal saline), then removed to 37 °C for 30 min. Also, the reaction was stopped in an ice water bath. Finally, the mixture was centrifuged at 3,000 rpm/min for 15 min at 4 °C to obtain the supernatant, and the OD value of the supernatant at the wavelength of 540 nm was measured with a spectrophotometer. The concentration of serum hemolysin was assessed with the HC_50_ value, which was calculated based on the following equation: HC_50_ = OD value of the sample/OD value of SRBC 50% hemolysis x dilution factor.

### Leukocyte count in the peripheral blood

The third batch of mice was used for the determination of organ indexes, serum albumin, interferon-γ (IFN-γ), tumor necrosis factor-α (TNF-α), interleukin (IL)-2, IL-6, IL-8, IL-1β, and leukocyte count as well as histomorphology. After the final administration for 30 min, the mice were anesthetized with 5% ether and the blood samples were collected by removing their eyeballs, and then 20 μl blood samples were put into 0.38 ml 2% acetic acid and mixed. The number of white blood cells in the peripheral blood was measured by a counting plate under a fluorescence microscope (Olympus, Tokyo, Japan).

### Serum chemistry and immunologic factors levels

The concentrations of serum albumin (ALB) were measured by using an Olympus AU400 Clinical Chemistry Analyzer (Olympus, Shizuoka, Japan). Serum levels of INF-γ, TNF-α, IL-2, IL-6, IL-8, IL-1β were measured by using specific enzyme-linked immunosorbent assay (ELISA) kits by following the instructions of the manufacturer and purchased from the Jiangsu MEIMIAN Industry Co., Ltd (Jiangsu, China).

### Hematoxylin and eosin stain staining

The tumor tissues of the mice were fixed in 10% formalin for observing general morphology and were prepared for 4 μm paraffin-based slices. After that, the slices were stained with hematoxylin and eosin (H&E), light field microscope was used to observe the tissue histological changes.

### TUNEL staining

The deparaffinized tumor tissue sections in each group were stained using a Colorimetric TUNEL Apoptosis Assay Kit (C1091, Beyotime Biotechnology, China) according to the manufacturer’s instructions.

### RNA extraction and quantitative real-time PCR (RT-PCR) analysis

Based on the manufacturer’s instructions, the total RNA of tumor tissues was isolated using TRIzol Reagent (Invitrogen, Carlsbad, CA, USA), and was quantified by NanoDrop spectrophotometer (ND-2000, NanoDrop Technologies, USA). Then, cDNA was synthesized using PrimeScript RT Master Mix (TaKaRa, Dalian, China). Subsequently, RT-PCR was conducted using a CFX96 Real-Time PCR Detection System (Bio-Rad, CA) with an SYBR Green PCR Master Mix (TaKaRa, Dalian, China) and the conditions were as follows: 95 °C for 10 min, followed by 95 °C for 15 s and 60 °C for 1 min (40 cycles). Glyceraldehyde 3-phosphate dehydrogenase (GAPDH) was used as an internal reference. The primer sequences of TLR4, MyD88, NF-κB, Wnt1, β-catenin, and GAPDH were obtained from Shanghai Sangon Company (Sangon, Shanghai, China) as follows: TLR4: forward 5′-TCTGCAATGTCTCTGGCAGG-3′, reverse 5′-CTGAGACTTGGTAGGGCCAC-3′; MyD88: forward 5′-CTAGGACAAACGCCGGAACT-3′, reverse 5′-ATTAGCTCGCTGGCAATGGA-3′; NF-κB: forward 5′-GCATCGTCCCAAAGGAGGAA-3′, reverse 5′-TCTGTGCGTGGCAACTACAT-3′; Wnt1: forward 5′-AACAGTAGTGGCCGATGGTG-3′, reverse 5′-GGGTTCTGTCGGATCAGTCG-3′, β-catenin: forward 5′-GGTGCTGACTTTGCTTGCTT-3′, reverse 5′-AGGCTACACAATGTTACACGTC-3′; GAPDH: forward 5′-TGTCAAGCTCATTTCCTGGTATG, reverse 5′-TTATGGGGGTCTGGGATGGA-3′. Finally, the relative expression of related-molecules was quantified by the 2^−ΔΔCt^ method [13].

### Faeces bacterial composition

To investigate the diversity of the intestinal microflora, the stool specimens were collected in the third batch of mice. DNA from stool samples was isolated using ZR Fecal DNA MiniPrep (Zymo Research, Irvine, CA) according to the manufacturer’s instructions, and the quality and quantity of the preparations were determined by NanoDrop spectrophotometry and stored at  − 20 °C. The bacterial composition of stool samples was explored for the following selected microbiota constituents: *total bacteria, Enterococcus, Enterobacteriaceae, Bifidobacterium spp., and Lactobacillus spp*. One μL of DNA (0.5 ng) was used in each 20 μL of 1 × SYBR-Green PCR Master Mix kit (Takara, Japan), and the real-time PCR was performed using an ABI 7500 real-time PCR system (Applied Biosystems, CA). The following primers were used in this study: primers for total bacteria detection, forward (5′-TCCTACGGGAGGCAGCAGT-3′); reverse (5′-GGACTACCAGGGTATCTAATCCTGTT-3′). primers for Enterococcus detection, forward (5′-AACCTACCCATCAGAGGG-3′); reverse (5′-GACGTTCAGTTACTAACG-3′). primers for Enterobacteriaceae detection, forward (5′-CATTGACGTTACCCGCAGAAGAAGC-3′); reverse (5′-CTCTACGAGACTCAAGCTTGC-3′); primers for Bifidobacterium spp., detection, forward (5′-GGGTGGTAATGCCGGATG-3′); reverse (5′-TAAGCCATGGACTTTCACACC-3′). primers for Lactobacillus spp., detection, forward (5′-AGCAGTAGGGAATCTTCCA-3′); reverse (5′-ATTTCACCGCTACACATG-3′). The primer sequences for GAPDH (internal control) are 5′-CCTCGTCTCATAGACAAGATGGT-3′(forward primer) and 5′-GGGTAGAGTCATACTGGAACATG-3′ (reverse primer). The 2^−ΔΔCt^ method was used to calculate the relative levels of selected intestinal microflora.

### Cell counting kit-8 (CCK-8) assay

SiHa cells were cultured and grown at a density of 1 × 10^4^ cells/well into 96-well plates, and treated with ICA at different concentrations (0, 10, 30, 90, 120, 150 μmol·L^−1^)for 24 h, 48 h, and 72 h, respectively, in a humidified incubator at 37 °C with 5% CO_2_. After incubation, 10 μL/well CCK-8 reagents (Beyotime, China) were added to the medium and then incubated for 2 h, and the optical density (OD) of every well was measured at 450 nm using a microplate reader (Thermo Fisher Scientific, Inc.). The experiments were repeated three times.

### Transwell assay

Transwell assay was used to assess the invasion ability of SiHa cells with ICA treatment. The matrigel mixture was added to the upper chamber (Costar, Cambridge, MA, USA) in each well and cultured at 37 °C for 1 h, SiHa cells (1 × 10^5^ cells) suspended in 200 μl serum-free DMEM were added to the upper chamber, and 600 μl of DMEM supplemented with 20% FBS was added to the lower chamber. To both chambers, 0, 30, 90, 120 μmol·L^−1^ of ICA was added. 48 h later, the chambers were fixed in formaldehyde (4%) and stained with 1% crystal violet. For migration assay, the chambers were changed into without matrigel chambers. We randomly selected 6 visual fields from each well and imaged them under an inverted microscope at 200 × magnification.

### Flow cytometry

To investigate the cell apoptosis, SiHa cells were incubated in six-well plates at a density of 3 × 10^5^ cells/well overnight, and then treated with ICA (0, 30, 90, 120 μmol/L) for 48 h. After treatment, SiHa cells were collected, washed with phosphate buffer saline (PBS, Gibco, USA), and digested by 0.25% trypsin, and dissociated into a single cell. Afterward, the cell suspension was mixed with 5 μL of Annexin V-FITC and 10 μL of propidium iodide (PI) for 10 min at room temperature in the dark according to the manufacturer’s protocol and analyzed using the FACS Calibur (BD Biosciences, USA).

### ELISA assay

SiHa cells were pre-treated with ICA (0, 30, 90, 120 μmol·L^−1^) for 1 h and were then exposed to lipopolysaccharide (LPS, 1 μg/mL, Sigma-Aldrich) for 24 h. The supernatants were collected and centrifuged at 3000 rpm/min for 15 min at 4 °C to remove cell debris. Then, the levels of TGF-β1, IL-17A, IL-10, IL-4, IL-6, and TNF-α in the culture medium were quantified by using commercially available ELISA kits (Excellbio, Shanghai, China) according to the manufacturer’s instructions.

### Western blot

SiHa cells were seeded on six-well plates at a density of 3 × 10^5^ cells/well overnight and then treated with different concentration of ICA (0, 30, 90, 120 μmol·L^−1^) for 24 h at 37 °C and 5% CO_2_. The total proteins of tumor tissues or SiHa cells were extracted by using RIPA lysis buffer (Beyotime), and the protein concentration was measured using the BCA Protein Assay Reagent (Beyotime). Protein (30 μg) was isolated using 8% ~ 12% sodium dodecyl sulfate–polyacrylamide gel (SDS-PAGE) and subsequently transferred to polyvinylidene difluoride (PVDF) membrane (Invitrogen, Thermo Fisher Scientific, Inc.). The PVDF membrane was blocked with 5% no-fat milk for 1 h at room temperature and then reacted with primary antibodies against TLR4 (1:1000, ab13556, Abcam, Cambridge, UK), MyD88 (1:2000, ab2064), NF-κB P65 (1:1000, ab207297), Wnt 1 (1:1000, ab15251), β-catenin (1:500, ab16051), P16(1:2000, ab51243), P53 (1:1000, ab26), Ki67 (1:2000, ab15580), Survivin (1:800, ab134170), Bax (1:1000, ab32503), Bcl-2 (1:2000, ab182858), c-Myc (1:1000, ab32072), E-cadherin (1:2000, ab40772), Vimentin (1:1000, ab92547), GAPDH (1:5000, ab8245), and β-actin (1:5000, ab179467) at 4 °C overnight. After washing with TBST, then, the membrane was exposed to the appropriate secondary antibody (1: 5000, Abcam) conjugated with horseradish peroxide at room temperature for 2 h. Finally, the PVDF membranes were incubated with enhanced chemiluminescence (ECL) detection reagents (Beyotime), and the relative expression was analyzed using Image J software (National Institutes of Health, Bethesda, MD, USA) normalized to β-actin or GAPDH.

### Statistical analysis

All data were presented as mean ± standard deviations (SD) and SPSS 22.0 software was used to perform the statistical analysis. Differences between two groups were analyzed with the Student’s t-test and one-way analysis of variance (ANOVA) was employed for comparisons among various groups. The statistical significance was set at *P* < *0.05*.

## Results

### ICA inhibited the growth of mice bearing U14 cervical tumor by inducing apoptosis in vivo

**Fig. 1:**
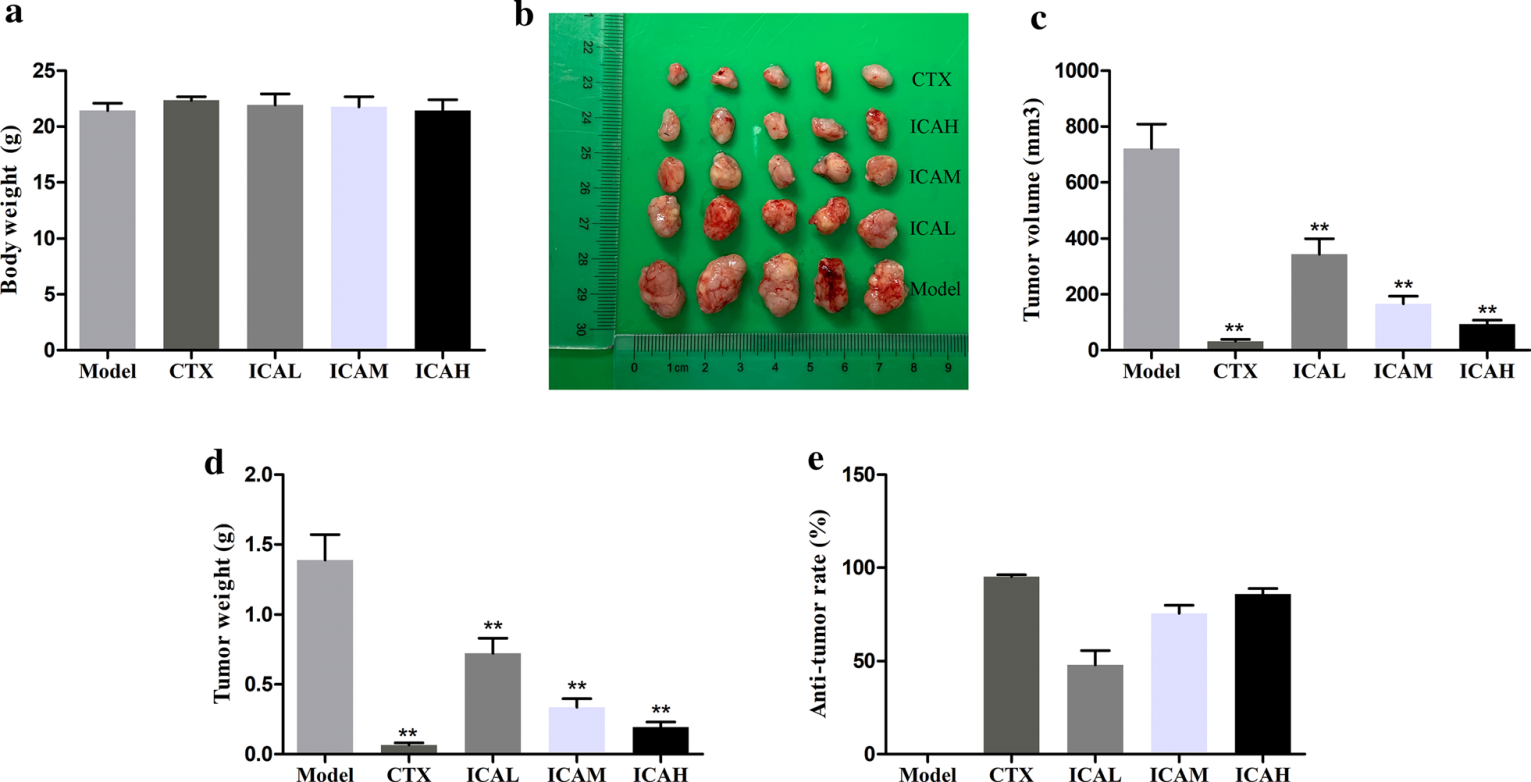
Effects of ICA on tumor growth in vivo**.** **a** The body weight of tumor-bearing mice after ICA treatment for 28 days. **b** The mice were sacrificed after 28 days of treatment and the tumor tissues were isolated and photoed. **c** Tumor volumes were calculated in each group, **P < 0.01 vs model group, n = 5. **d** Tumor weights were quantified in each group. **P < 0.01 vs model group, n = 5. **e** The anti-tumor rate was calculated as follows: (1−average weight of tumor in treatment group/average weight of tumor in model group) × 100%

**Fig. 2:**
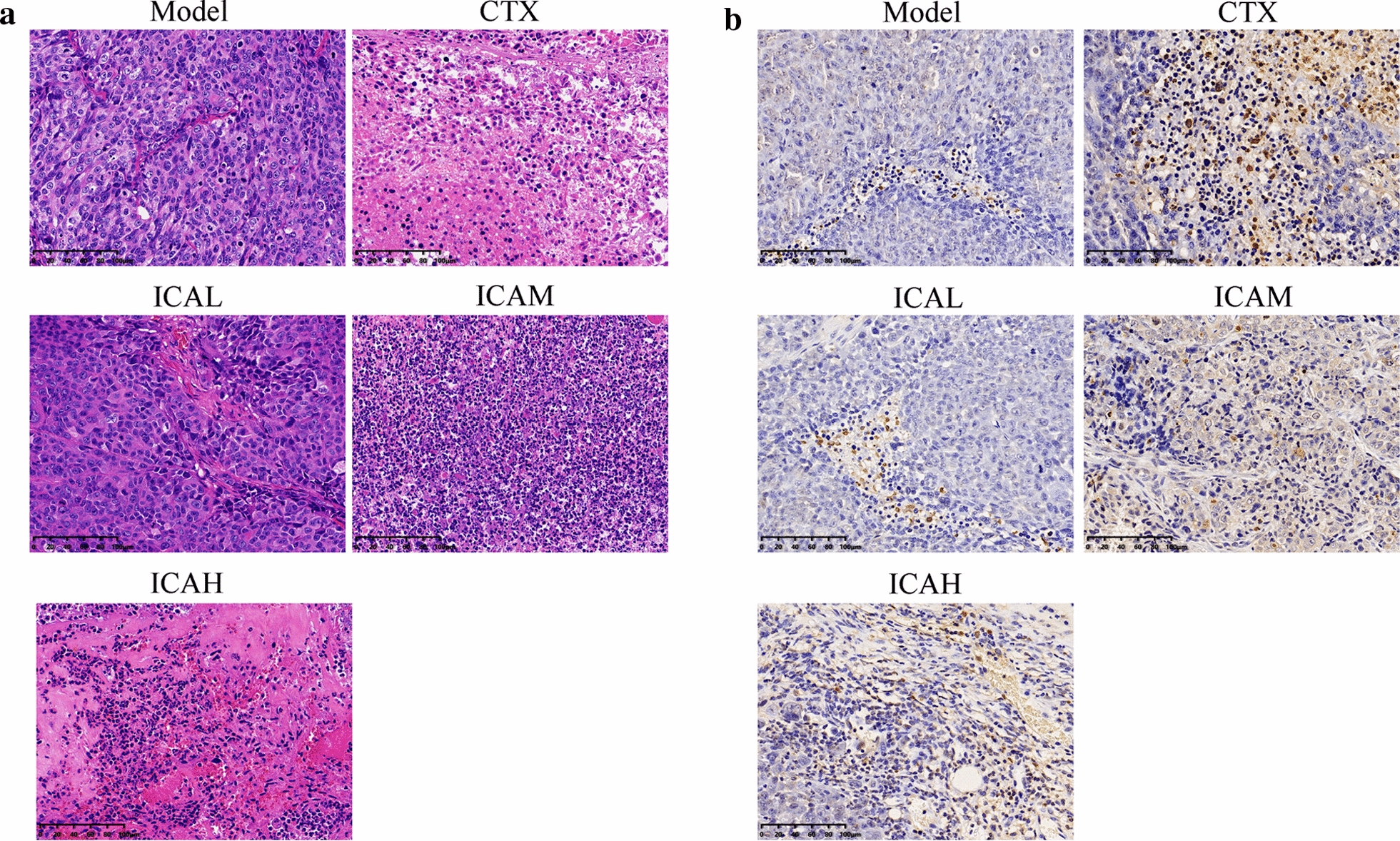
Inhibition of tumor growth in U14 tumor-bearing mice via inducing apoptosis by ICA. **a** Representative histological analysis of tumor tissues of U14 tumor-bearing mice (magnification, × 200). **b** TUNEL staining of tumor tissues in each group. The magnification is 200 × . The scale bar is 100 μm

To study the antitumor effects of ICA on cervical cancer in vivo, a U14 tumor-bearing xenograft was used. In the present study, we demonstrated that there were no statistical differences in body weight of tumor-bearing mice that were fed with ICA (Fig. 1a). In addition, as shown in Fig. 1b–e, both CTX and ICA significantly inhibited tumor growth compared with the model group. The present study showed that no significant necrosis was observed in the histological examination of tumor tissues by HE staining in the model group, but the CTX and ICA-treated groups tumors presented a cell layer loosening, nuclear condensation or fragmentation of tumor cells, and extracellular space dilatation (Fig. 2a). Furthermore, the results of TUNEL confirmed that either CTX or ICAM/ICAH resulted in marked apoptosis in tumor tissues compared with the model group (Fig. 2b). These results revealed that ICA suppressed the cervical tumor growth in vivo with a dose-dependent relationship.

### ICA improved the macrophage phagocytosis and humoral immune function of tumor-bearing mice

**Fig. 3:**
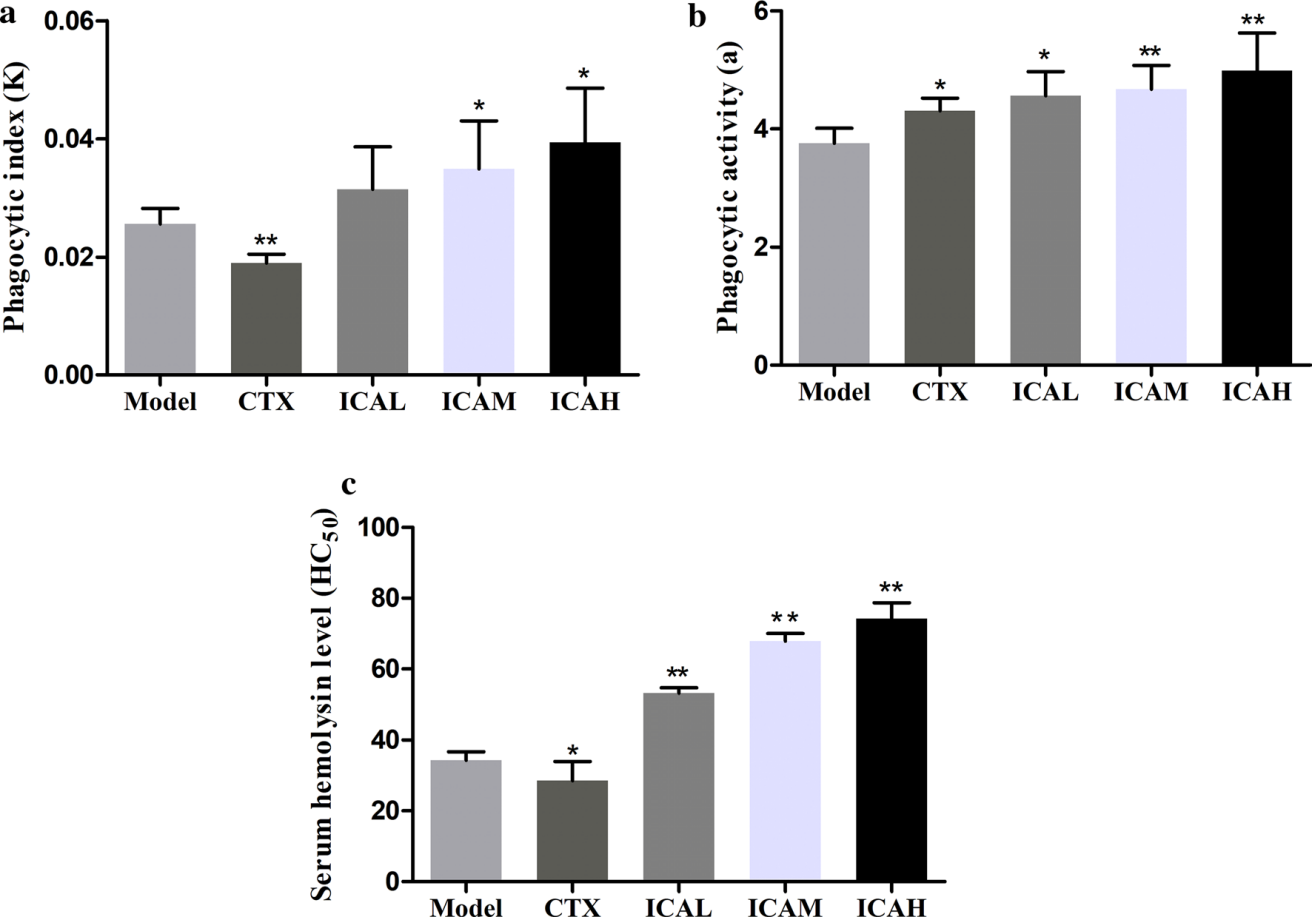
Effects of ICA on the humoral immune function in cervical tumor mice. **a** The phagocytic index and **b** phagocytic and activity of the carbon clearance test. **c** The serum hemolysin level in tumor-bearing mice with ICA treatment. Values are expressed as the mean ± standard deviation. ^*^P < *0.05*; ^**^P < *0.01 vs* model group, n = 5

As illuminated in Fig. 3a, b, compared with that in the model group, the phagocytic index and activity in the ICR-treated group were significantly increased. Additionally, compared with that in the model group, the HC_50_ value in the CTX group was significantly decreased, while the HC50 values in the ICR-treated groups had an increasing trend in a concentration-dependent manner, as presented in Fig. 3c, indicting that ICA significantly increased the serum hemolysin level to ameliorate the humoral immune function in cervical tumor mice.

### ICA improved the spleen and thymus indexes, serum ALB levels, and leukocytes count of cervical cancer-bearing mice

**Fig. 4:**
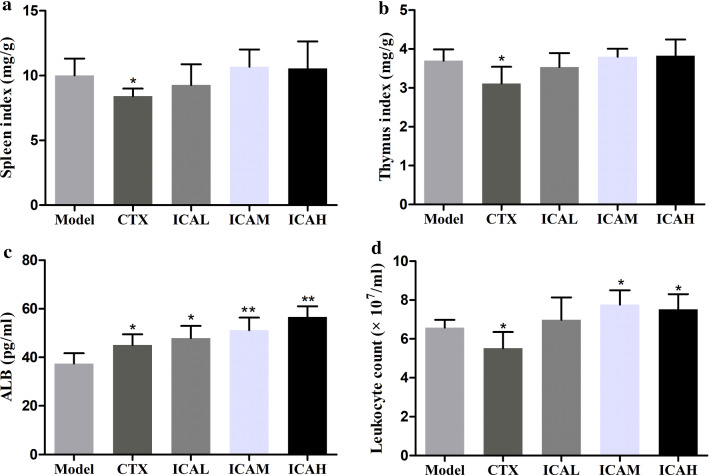
Effects of ICA on **a** the spleen index, **b** thymus index, **c** serum ALB level, and **d** leukocyte count in peripheral blood. Values are expressed as mean with SD. ^*^P < *0.05*; ^**^P < *0.01 vs* model group, n = 5

To further assess the enhanced immunity effect of ICA in vivo, the spleen and thymus indexes were calculated, and results revealed that spleen and thymus indexes were higher in the ICA-treated group compared to the CTX group (Fig. 4a, b). No obvious differences in terms of spleen and thymus indexes were found among the model, ICAL, ICAM, and ICAH groups. Also, the levels of serum ALB were significantly increased by CTX and ICA administrating compared with those of model group (Fig. 4c). Moreover, compared with that in the model group, we found that the number of leukocytes in the CTX group was significantly decreased, whereas the number of leukocytes in the ICA-treated groups was significantly elevated, especially in the ICAM and ICAH group (Fig. 4d).

### ICA improved the activity of immune prevention of cervical cancer-bearing mice

**Fig. 5:**
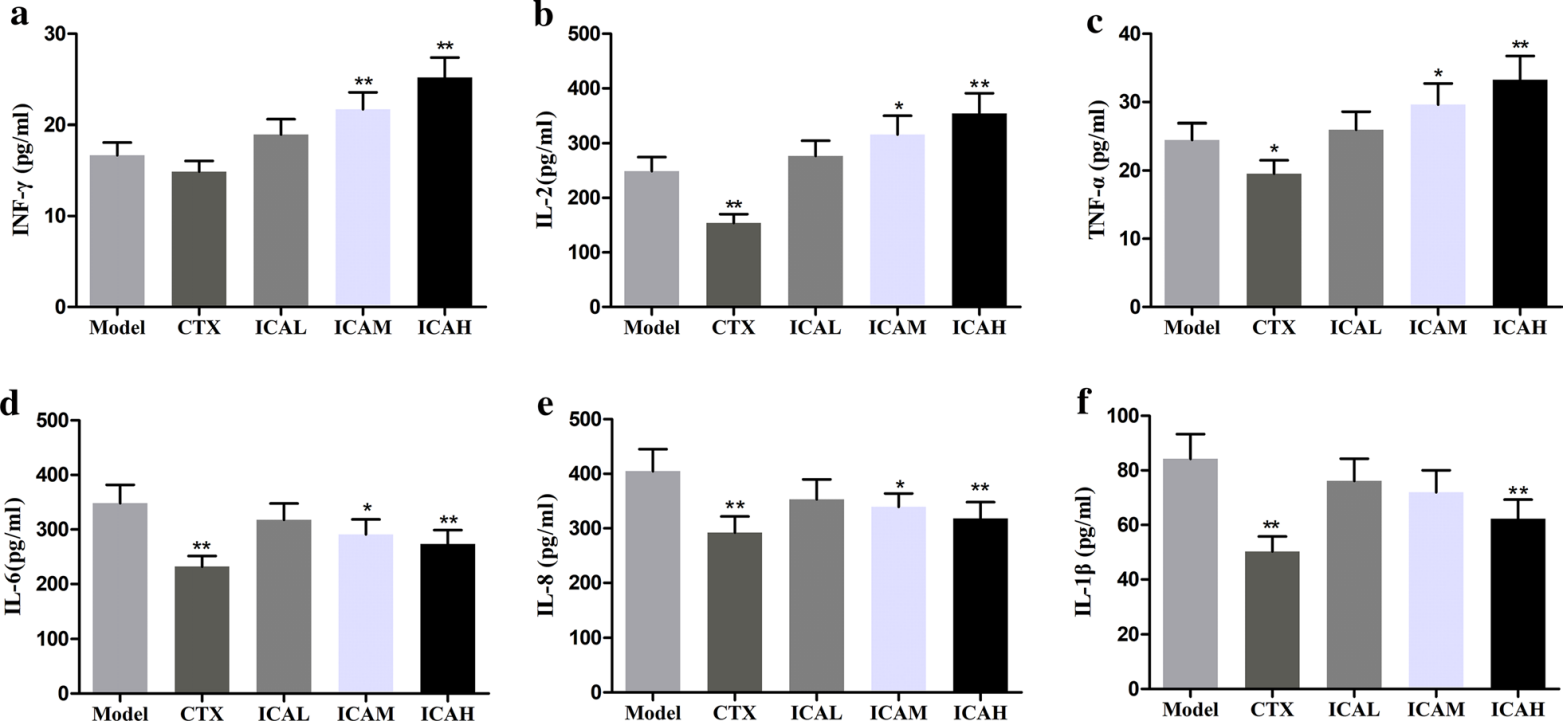
Effect of ICA on serum **a** IFN-γ, **b** IL-2, **c** TNF-α, **d** IL-6, **e** IL-8, and **f** IL-1β levels in tumor-bearing mice. Data are shown as means ± SD., n = 5. ^*^P < *0.05*; ^**^P < *0.01* as compared with the model group

Comparing with the model group, the activity of immune defenses of cervical cancer-bearing mice in ICA-treated groups was observably enhanced. Mice treated with ICA in each group had a prominent improvement in the levels of serum IFN-γ, IL-2, and TNF-α with a certain dose dependence (Fig. 5a–c). And, the expression levels of serum IL-6, IL-8, and IL-1β were lower than those in the model group (Fig. 5d–f). These results suggest that ICA could ameliorate the immune defense functions of U14 tumor-bearing mice.

### ICA treatment improved the intestinal flora disorders in cervical cancer-bearing mice

**Fig. 6:**
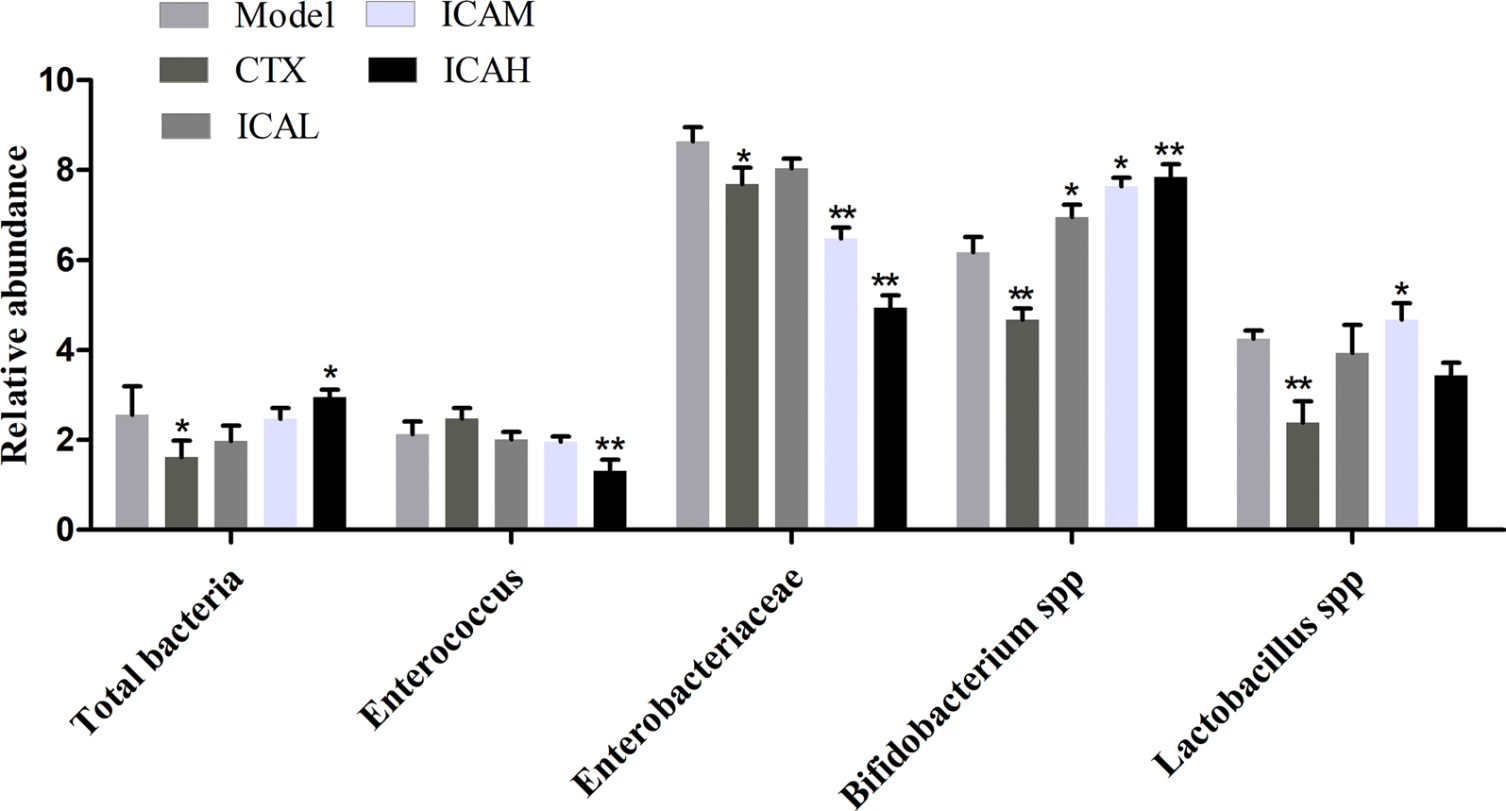
Quantification of dominant bacterial in stool samples of the U14 tumor-bearing mice

Previous evidence found that the gut microbiome is proposed to alter host immunity by modulating multiple immunologic pathways in cervical cancer [14]. To investigate whether the intestinal microflora play roles in the efficacy of ICA, the abundance of *total bacteria, Enterococcus, Enterobacteriaceae, Bifidobacterium spp, and Lactobacillus spp* was examined in stool samples from each group by qPCR. As illustrated in Fig. 6, we found that the levels of *total bacteria*, *Bifidobacterium spp*, and *Lactobacillus spp* were significantly increased in ICA-treated groups compared to those in the model group. In contrast, the levels of *Enterococcus* and *Enterobacteriaceae* were lower in ICAH samples than in the model group. These results indicating that the antitumor effects of ICA treatment may be due to affect the intestinal microflora in cervical cancer.

### ICA effectively suppressed cell proliferation in mice model

**Fig. 7:**
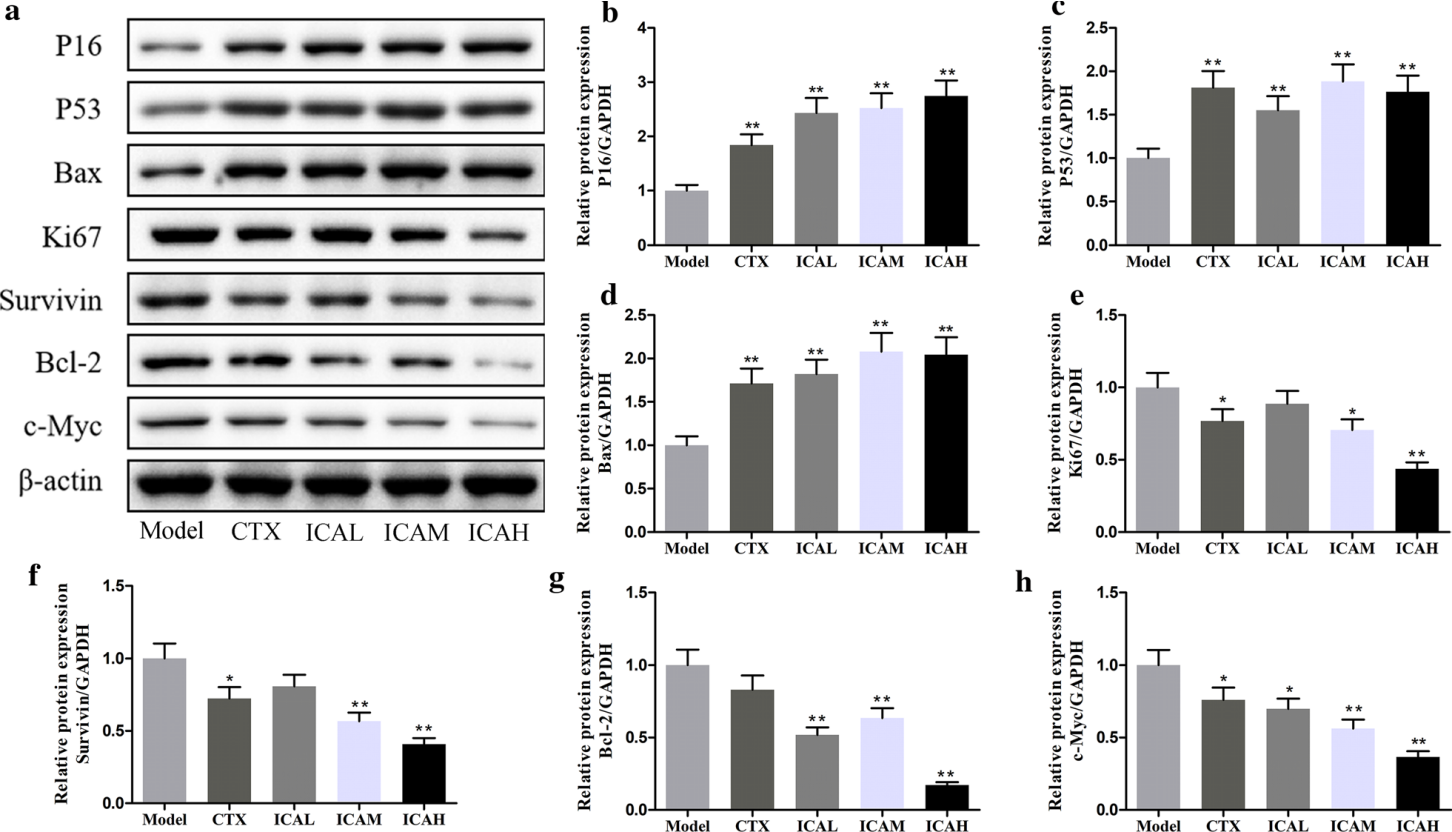
Effect of ICA on cell proliferation-related proteins in U14 tumor-bearing mice model. **a** Western blot analysis of P16, P53, Bax, Ki67, survivin, Bcl-2, and c-Myc proteins in tumor tissues of U14 tumor-bearing mice model that treated with CTX or ICA. **b–d** CTX and ICA increased P16, P53, and Bax protein expressions in tumor tissues in a dose-dependent manner. **e–h** Treating with ICA dose-dependently reduced Ki67, survivin, Bcl-2, and c-Myc levels in mice models. Results are means ± SD, n = 5; ^*^P < *0.05*; ^**^P < *0.01 vs* model group

**Fig. 8:**
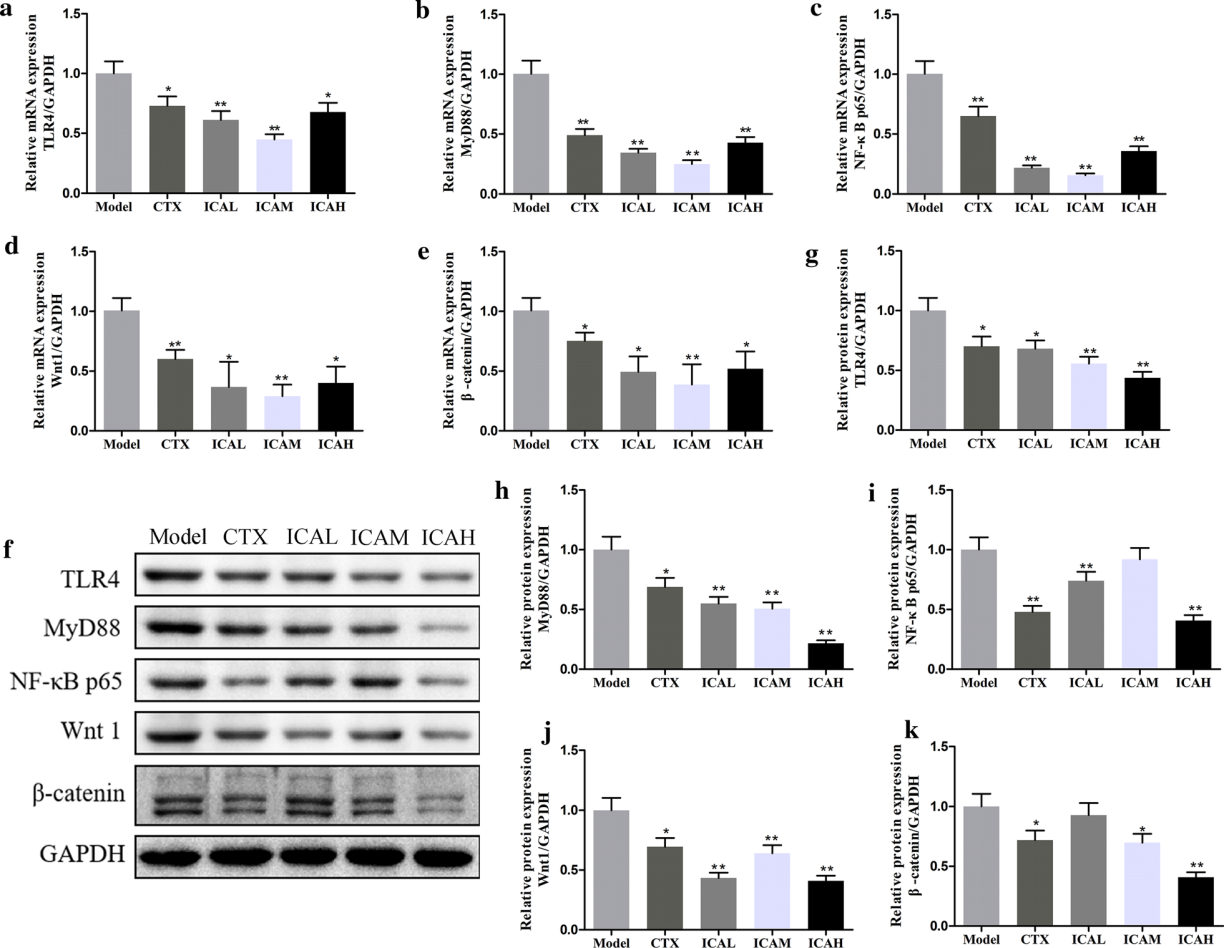
Effect of ICA on TLR4/MyD88/NF-κB and Wnt/β-catenin signaling pathways in the xenograft tumor tissues. **a–e** The mRNA expression level of TLR4, MyD88, NF-κB p65, Wnt 1, and β-catenin was detected by quantitative real-time PCR. **f** Representative images of protein bands and protein **g–k** of TLR4, MyD88, NF-κB p65, Wnt 1, and β-catenin expression levels. Data are presented as the means ± SD, n = 5; ^*^P < *0.05*; ^**^P < *0.01 vs* model group

It is generally appreciated that the unlimited cell proliferation was an important property characteristic of malignancy, thereby we verify the expression of P16, P53, Bax, Ki67, survivin, Bcl-2, and c-Myc under the presence of CTX and ICA in tumor tissues of mice model by western blot. As shown in Fig. 7a–d, the protein expression levels of P16, P53, and Bax were increased in a dose-dependent manner by ICA treatment. Moreover, the results revealed that the Ki67, survivin, Bcl-2, and c-Myc expression levels all decreased in the CTX and ICA-treated groups (Fig. 7e–h). We also measured TLR4, MyD88, NF-κB p65, Wnt 1, and β-catenin mRNA and protein expression in the xenograft tumor tissues. Compared to the model group, the mRNA(Fig. 8a–e) and protein (Fig. 8f–k) expression levels of TLR4, MyD88, NF-κB p65, Wnt 1, and β-catenin all decreased in CTX, ICAL, ICAM, and ICAH groups, respectively. Therefore, the ICA may be an effective drug to attenuate the TLR4/MyD88/NF-κB and Wnt/β-catenin signaling pathways to suppress tumor cell proliferation in xenograft in mice models.

### ICA inhibits the migration, invasion, the levels of immunosuppressive factors, and promoted the apoptosis of SiHa cells

**Fig. 9:**
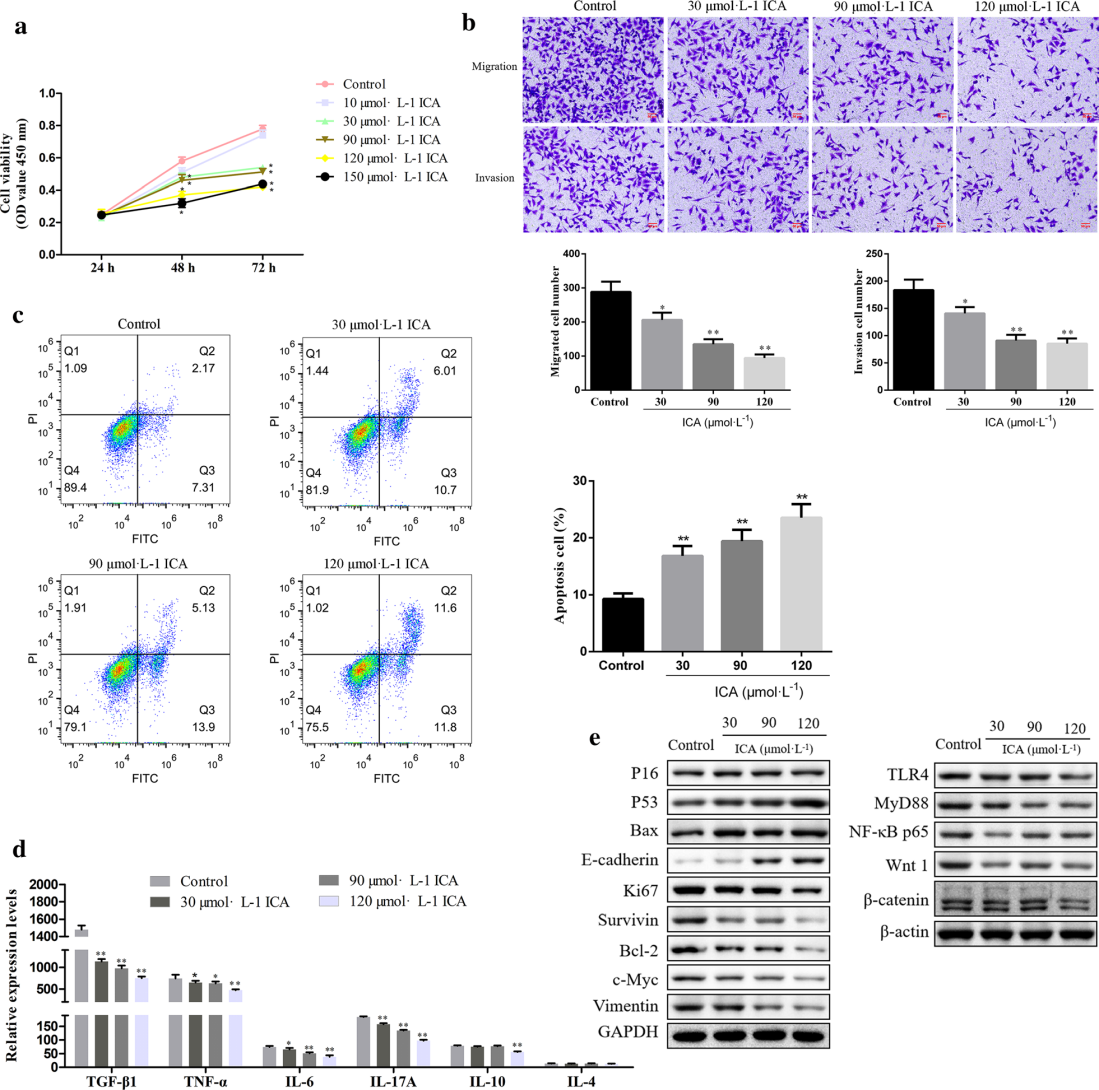
Effects of ICA on the migration, invasion, and apoptosis of SiHa cells. **a** CCK-8 assays were performed to demonstrate cell viability in ICA-treated SiHa cells. **b** Transwell assays were performed to examine the effects of ICA on SiHa cells migration and invasion. **c** SiHa cell apoptosis was determined by flow cytometer analysis after ICA treatment. **d** The expression levels of immunosuppressive factors were determined by ELISA assay. **e** Western blotting was performed to detect the expression levels of TLR4, MyD88, NF-κB p65, Wnt 1 β-catenin, P16, P53, Ki67, Survivin, Bax, Bcl-2, c-Myc, E-cadherin, and Vimentin in ICA-treated SiHa cells. Data are expressed as the mean ± SD. ^*^P < *0.05*; ^**^P < *0.01 vs* control group

To evaluate the functional roles of ICA in the cervical cancer cell, we treated the SiHa cells with increasing concentrations of ICA. To begin with, we estimated the cell viability by using CCK-8 assays for 24 h, 48 h, and 72 h. As demonstrated in Fig. 9a, ICA suppressed the cell viability of SiHa cells in a dose-dependent way. Moreover, transwell assays were conducted to assess the effect of ICA on SiHa cell's motility. As shown by Fig. 9b, the number of cell migration and invasion was significantly reduced in the ICA-treated groups (30, 90, 120 μmol·L^−1^) compared with that in the control group. In addition, ICA promoted the apoptosis of SiHa cells (Fig. 9c). We further determined that the levels of immunosuppressive factors of LPS-mediated SiHa cells. As a result, ICA dramatically inhibited the expression levels of TGF-β1, TNF-α, IL-6, L-17A, and IL-10; Also, there was no significant difference in expression levels of IL-4 between ICA-treated groups and control group (Fig. 9d). Furthermore, western blot assays were determined to measure the protein expression of cell proliferation-/EMT-representative markers, respectively. As shown in Fig. 9e, the western blot results showed that the protein expression levels of P16, P53, Bax, and E-cadherin in ICA groups were significantly higher than those in the control group. Meanwhile, the protein expression of Ki67, survivin, Bcl-2, c-Myc, Vimentin, TLR4, MyD88, NF-κB p65, Wnt 1, and β-catenin in SiHa cells treated with ICA was significantly lower than in the control group. These findings suggesting that ICA had attenuated migration and invasion capacities, as well as promoting the apoptosis of SiHa cells via modulating the TLR4/MyD88/NF-κB and Wnt/β-catenin signaling pathways.

## Discussion

Cervical cancer is the fourth most common cancer in women and the malignancy results in over 311 000 deaths in 2018 [15]. It is estimated that approximately 90% of cervical cancers occur in some developing countries that a deficiency of cancer screening programs and HPV vaccination programs [16]. Cervical cancer is largely preventable. Currently, immunotherapy is the most effective cancer treatment strategy in recent years and has improved the quality of life of patients in the clinic. Fortunately, three types of prophylactic vaccines, quadrivalent HPV vaccine, bivalent HPV vaccine, and a new nonavalent HPV vaccine, are commercially available and that protecting against 90% of HPV infection [17]. However, these vaccines difficult to eliminate pre-existing HPV infections. And, due to women with the metastasis and recurrence of cervical cancer, the overall prognosis remains poor. Therefore, it is significant to explore and develop more promising therapeutic agents in order to further improve the quality of life of cervical cancer patients.

Icariin (ICA), a natural flavonoid glucoside, is isolated from the dry stems and leaves of genus *epimedium* and has shown a range of pharmacological activities, including immunological regulation, anti-inflammatory effects, and anticancer potency [18]. Recently, Hao et al. observed that ICA can significantly induce antitumor immunity in murine B16F10 melanoma and MC38 colorectal tumors mice in a CD8 T-cell-dependent way [19]. In addition, ICA has been demonstrated to attenuate the growth and invasion ability of Human oral squamous cell carcinoma in vitro and in vivo by inducing the down-regulation of Toll-Like Receptor 4 (TLR4)/ NF-κB pathway [20]. Also, Huang et al. demonstrated that ICA inhibited the growth of HeLa cervical cancer cells dose-dependently via inhibiting the mTOR/PI3K/AKT signaling cascade [21]. Despite many reports about the anticancer activities of ICA, little is known about the effect of ICA on U14 tumor-bearing mice and SiHa human cervical cancer cells. And, the mechanisms underlying the anti-tumor effect of ICA on cervical cancer have not yet been illuminated. Thus, this study aimed to investigate the effects and mechanisms of ICA on cervical cancer in vivo and in vitro.

In the current study, U14 and SiHa cells were employed to investigate the therapeutic effects of ICA on cervical cancer in vivo and in vitro, respectively. As a result, we found that ICA observably inhibited the growth of subcutaneous tumors due to the induction of apoptosis. Also, the carbon clearance test was often been represented for the phagocytic activity of macrophages [22], and the results suggested that ICA treatment resulted in a significant increase of the phagocytic capacity in U14 tumor-bearing mice. Serum hemolysin was produced by B lymphocytes by touching with red cells antigens, hemolyzes red blood cells, which also represents the proliferation and differentiation of B cells [23]. An earlier study has shown that the levels of serum hemolysin in the SRBC‑immunized animals reflect the function of humoral immunity [23, 24]. Similarly, the results of the serum hemolysin test in this study revealed that ICA was capable to enhance the humoral immune function in U14 tumor-bearing mice in a dose-dependent manner. The development, differentiation, and maturity of T lymphocytes are associated with the thymus tissues and the thymus index on behalf of the weight of the thymus [25]. In addition, the spleen is a peripheral immune organ that is related to the immune response and activation, and immune cell differentiation and proliferation also markedly increased its weight [26]. Hence, a reduction of spleen and thymus weight indicated that the declined immune function of animals. Moreover, leukocytes play an important role in the body's defense system, and it is an important indicator to assess the immune function by counting the number of leukocytes. In this study, we showed that ICA treatment was significantly upregulated the spleen and thymus index, as well as the levels of ALB in U14 cervical cancer mouse models. Furthermore, several studies have shown that cytokines can have quite an interesting effect on tumor immunity [27, 28]. IFN‑γ, a multifunctional small‑molecule soluble protein, is produced by monocytes and lymphocytes [29], which plays an important role in the antiviral, anti-proliferative, and anti-tumor in cervical cancer [30]. IL-2 has been used for the treatment of many different kinds of cancer, and Lagunas-Cruz et al. reported that IL-2 decreases cervical cancer cell proliferation through the transient arrest of the G1 phase [31]. TNF‑α, a cytokine naturally synthesized by the macrophages that directly leads to the death of cancer cells [32]. Meanwhile, the inflammatory cytokines, such as IL-6, IL-8, and IL-1β can promote tumor proliferation and the occurrence and development of cervical cancer [28]. In our study, we showed that the expression levels of IFN‑γ, IL-2, and TNF‑α were higher in ICA-treated groups than in the CTX group to ameliorate the immune function of mice. Besides, ICA also inhibited the IL-6, IL-8, and IL-1β expression. These results had demonstrated that ICA significantly inhibited tumor growth in cervical cancer by inducing apoptosis and increasing the body's immunological function.

A number of studies have found that gut microbial composition has been implicated in cervical tumorigenesis [14, 33]. In this study, our research showed that dysbiosis in U14 tumor-bearing mice. We showed that the total concentration of bacterial DNA was higher in the ICAH group. Interestingly, the characteristic microbiome was differentiated significantly in relative abundance between ICA groups and model group, including *Enterococcus, Enterobacteriaceae, Bifidobacterium spp., and Lactobacillus spp*. *Enterococcus* and *Enterobacteriaceae* populations were significantly decreased in the ICA group compared to model controls, while *Bifidobacterium spp., and Lactobacillus spp* were significantly enriched in ICA-treated groups. According to the study, the antitumor effects of ICA may benefit from it was significantly altered diversity and composition of the gut microbiota, and these findings provide a rationale for further study of the gut microbiome in cervical cancer with ICA treatment.

Next, we sought to discover whether ICA inhibits cervical cancer progression through TLR4/MyD88/NF-κB and Wnt/β-catenin signaling pathways in vivo and in vitro. Results showed that ICA inhibited the cell viability of SiHa cells in a dose-dependent manner. In addition, we found that ICA inhibited cervical cancer SiHa cells proliferation, migration, invasion, and the expression levels of TGF-β1, TNF-α, IL-6, IL-17A, and IL-10, as well as promoted cell apoptosis. And, we do observe that ICA promotes the protein expression levels of P16, P53, Bax, E-cadherin, and also inhibited Ki67, survivin, Bcl-2, c-Myc, and vimentin expression. Previous studies have shown that TLR4/MyD88/NF-κB pathway is linked to the inflammatory response in hepatocellular carcinoma [34], breast cancer [35], and tumor-associated macrophages (TAMs) on Ishikawa cells [36]. Additionally, Ghasemi et al., demonstrated that the inhibition of NF-kB and Wnt/β-catenin pathways were beneficial to inhibit the invasion and proliferation of cervical cancer cells [37]. In the present study, ICA has remarkably reduced the expression levels of TLR4, MyD88, NF-κB p65, Wnt 1, and β-catenin in cervical cancer SiHa cells. Our finding is consistent with predecessor's studies and confirmed that ICA suppressed the tumor progression of cervical cancer by inhibiting TLR4/MyD88/NF-κB and Wnt/β-catenin pathways in vitro and in vivo. However, there are still some limitations that exist in this study. First, the enhanced immunity effect of ICA should be further confirmed with different immune cells by using flow cytometry. Second, although the TLR4 levels have been verified in this study, the roles of TLR4 are still to be investigated. We will further explore this topic in our future research by using TLR4 knockdown SiHa cells, U14 cells, and animals.

## Conclusion

Overall, we showed that icariin efficiently inhibits the inflammation, proliferation, invasion, as well as promotes apoptosis and immunity in cervical cancer by inhibiting TLR4/MyD88/NF-κB and Wnt/β-catenin pathways. Thus, it is expected that icariin could be used to treat cervical cancer as a valuable complementary therapy. However, more investigations are required to investigate the clinical effects and more accurate molecular mechanisms.

## Data Availability

All the data were available on the manuscript or from the corresponding author on reasonable request.

## Abbreviations

ICA: Cariin
HPV: Human papillomavirus
TCM: Traditional Chinese medicine
TME: Tumor microenvironment
TLRs: Toll-like receptors
NF-κB: Nuclear factor-kappa B
ATCC: American type culture collection
FBS: Fetal bovine serum
CTX: Cyclophosphamide
IFN-γ: Interferon-γ
TNF-α: Tumor necrosis factor-α
IL: Interleukin
ALB: Albumin
ELISA: Enzyme-linked immunosorbent assay
H&E: Hematoxylin and eosin
CCK-8: Cell counting kit-8
LPS: Lipopolysaccharide
SDS-PAGE: Sodium dodecyl sulfate–polyacrylamide gel
PVDF: Polyvinylidene difluoride
